# Teleneurorehabilitation in the COVID-19 Era: What Are We Doing Now and What Will We Do Next?

**DOI:** 10.3390/medsci9010015

**Published:** 2021-02-24

**Authors:** Rocco Salvatore Calabrò

**Affiliations:** IRCCS Centro Neurolesi Bonino Pulejo, S.S. 113, Contrada Casazza, 98124 Messina, Italy; salbro77@tiscali.it; Tel.: +39-090-60128840; Fax: +39-090-60128950

Dear Editor,

COVID-19 is an acute respiratory illness caused by the SARS-COV2, whose transmission occurs via droplets or direct contact with contaminated objects (i.e., fomites). Thus, the first-line preventive strategies are to increase the social distances, and potentiate all general prevention measures regarding the common infective respiratory diseases [1].

COVID-19 has enveloped the world causing greater morbidity and mortality to the most vulnerable populations, including older people and those with neurological disabilities [2].

Thus, the disease has had many impacts on people around the world including, among other issues, the need to stay at home, avoid public gatherings, wear masks and gloves. Notably, these issues are more difficult for persons with neurological disabilities who are the most vulnerable because of the altered mobility and cognitive dysfunction, the need for assistance for self-care, assistive devices, and supplies [1,2].

The unfolding COVID-19 pandemic is transforming neurological care more than any other crisis in modern history. Social distancing and quarantine have cut off access to routine medical care for numerous individuals with neurological diseases [3,4]. Indeed, many neurological patients are at increased risk when coinfected with COVID-19 because of their advanced age (e.g, patients with Alzheimer disease and Parkinson Disease), comorbid conditions (e.g., individuals with respiratory impairment in amyotrophic lateral sclerosis), or immunosuppressive treatments (e.g., people with multiple sclerosis). Besides routine visits and pharmacological treatments, neurological patients need neurorehabilitation due to their, often severe, motor and cognitive/behavioral disabilities.

To face the pandemic, the healthcare system had to rapidly change its organization worldwide. Indeed, many wards dedicated to chronic diseases and/or rehabilitation have been converted into acute ones to better deal with medical and neurological complications of patients affected by COVID-19 [5,6,7]. Early discharge from rehabilitation units of negative patients was performed, with shortening of their rehabilitation plan. Moreover, new admissions to neurorehabilitation have been suspended or temporarily reduced, as well as outpatient services. Thus, other ways to provide patients with the due rehabilitation/support were necessary [5].

Telemedicine is a means of providing health care services at distance by using ICT, and its use is particularly important during this COVID-19 pandemic. Potentially, every field of medicine may benefit from this tool, including telecardiology, teledermatology, telestroke and telerehabilitation [8]. The latter represents an emerging and innovative approach during the patient’s rehabilitation pathway helping in improving motor, cognitive or psycho-behavioral disorders. Indeed, teleneurorehabilitation may provide many types of interventions, including physiotherapy, speech, cognitive and behavioral therapy, occupational therapy, telemonitoring, and teleconsultation [9,10].

This is the reason why the tool can guarantee the continuity of care over time (as it can be immediately applied after discharge) and in space (by closing the gap between the hospital and patients’ home, also when they live far or in rural areas provided that internet connection is present). Moreover, teleneurorehabilitation can provide substantial cost savings (due to the reduction in specialized human resources and avoiding travel), improvements in comfort and patient lifestyle, and increased frequency and adherence to therapy [8].

Possible reasons for the use of telemedicine in the neurology field may include for weaning down of pain medicine, adjusting medications for spasticity or neuropathic pain, reevaluation of a decubitus or for reevaluation of how newly initiated medication regiments are working [11]. The tool can also be used to evaluate the performance and efficacy of neurological therapy. Moreover, physiatrists/neurologists as well as other rehabilitation professionals might use teleconferencing to bridge the gap between the acute care and rehabilitation hospital, for traditional inpatient team meetings and for meetings with home care therapists, especially during pandemics [8]. Specialty services can also be provided, such as monitoring control of glucose levels, blood pressure as well as monitoring the status of other vital signs, such as measurement of pulse oximetry, heart rate and weight. Finally, the tool could be used to reach out to children with developmental disabilities as to configure a family-centered and home-based models of care, in which digital physical therapy will adopt a modified framework of care [12].

Growing evidence supports the feasibility and effectiveness of such remote care delivery [8]. For example, remote care by a neurologist via videoconferencing was associated with outcomes comparable with regular outpatient visits, but with much greater efficiency [7]. Moreover, telemedicine can be used to deliver home-based interventions, such as activity-based training for survivors of stroke, which was as effective when delivered via telemedicine as through in-clinic programs [7,11].

Nonetheless, only few studies have investigated the role of neurorehabilitation during the COVID-19 pandemic [13,14,15,16]. In particular, the HomeCoRe (i.e., Home Cognitive Rehabilitation software) seems to offer an innovative approach and a valid support for home-based cognitive rehabilitation in neurodegenerative diseases, such as mild cognitive impairment and early dementia [13]. Moreover, another study found that a home care system using a commercial smartwatch and smartphone app equipped with machine learning can facilitate participation in home training and improve the upper limb function in patients with chronic stroke [15].

The rapid expansion of telemedicine into current daily practice also comes with challenges. One concern is about privacy and security, given that not all available tools for videoconferencing comply with internationally accepted standards to protect patient’s confidentiality. Another drawback is the potential to disrupt the traditional doctor–patient relationship, as the treatment of disease has long been viewed as a holistic process, with human contact, personal interaction, and direct communication valued as critical components of effective and compassionate care.

Finally, lack of reimbursement has also impeded a wider introduction of telemedicine services for those with private or no insurance, and only a few countries are addressing the critical legal and financial issues and standards of care [8].

The future course of the COVID-19 pandemic cannot be predicted with certainty, but teleneurorehabilitation will probably be an integral part of patient care for at least another year, and this change in care may become entrenched for the long term. Indeed, telerehabilitation not only decreases non-essential face-to-face training, but also provides earlier access to specialized care, reduces the burden of patient transport, and is often more comfortable for both patients and caregivers, avoiding the potential spread of COVID-19 during this pandemic. Incorporating telemedicine into the infrastructure of patient care will ensure a more viable and robust system that can withstand future global pandemics, or future waves of the current one.

Thus, the use of teleneurorehabilitation should become part of the new normal rather than the exception, encouraging the support and creation of innovative technology, even after this terrible pandemic ends.

## References

[1] Ashraf O., Virani A., Cheema T. (2021). COVID-19: An Update on the Epidemiological, Clinical, Preventive, and Therapeutic Management of 2019 Novel Coronavirus Disease. Crit. Care Nurs. Q..

[2] Smorenberg A., Peters E.J., van Daele P., Nossent E.J., Muller M. (2021). How does SARS-CoV-2 targets the elderly patients? A review on potential mechanisms increasing disease severity. Eur. J. Intern. Med..

[3] Roy B., Nowak R.J., Roda R., Khokhar B., Patwa H.S., Lloyd T., Rutkove S.B. (2020). Teleneurology during the COVID-19 pandemic: A step forward in modernizing medical care. J. Neurol. Sci..

[4] Nuara A., Fabbri-Destro M., Scalona E., Lenzi S.E., Rizzolatti G., Avanzini P. (2021). Telerehabilitation in response to constrained physical distance: An opportunity to rethink neurorehabilitative routines. J. Neurol..

[5] Calabrò R.S., Manuli A., Naro A., Rao G. (2020). How Covid 19 has changed Neurorehabilitation in Italy: A critical appraisal. Acta Biomed..

[6] Ganapathy K. (2020). Telemedicine and Neurological Practice in the COVID-19 Era. Neurol. India.

[7] Jodice F., Romoli M., Giometto B., Clerico M., Tedeschi G., Bonavita S., Leocani L., Lavorgna L. (2021). Digital Technologies, Web and Social Media Study Group of the Italian Society of Neurology. Stroke and digital technology: A wake-up call from COVID-19 pandemic. Neurol. Sci..

[8] Maresca G., Maggio M.G., De Luca R., Manuli A., Tonin P., Pignolo L., Calabrò R.S. (2020). Tele-Neuro-Rehabilitation in Italy: State of the Art and Future Perspectives. Front. Neurol..

[9] Maggio M.G., De Luca R., Manuli A., Calabrò R.S. (2020). The five ‘W’ of cognitive telerehabilitation in the Covid-19 era. Expert Rev. Med. Devices.

[10] De Luca R., Calabrò R.S. (2020). How the COVID-19 Pandemic is Changing Mental Health Disease Management: The Growing Need of Telecounseling in Italy. Innov. Clin. Neurosci..

[11] Cox N.S., Scrivener K., Holland A.E., Jolliffe L., Wighton A., Nelson S., McCredie L., Lannin N.A. (2021). A brief intervention to support implementation of telerehabilitation by community rehabilitation services during COVID-19: A feasibility study. Arch. Phys. Med. Rehabil..

[12] Rao P.T. (2021). A Paradigm Shift in the Delivery of Physical Therapy Services for Children with Disabilities in the Time of the COVID-19 Pandemic. Phys. Ther..

[13] Bernini S., Stasolla F., Panzarasa S., Quaglini S., Sinforiani E., Sandrini G., Vecchi T., Tassorelli C., Bottiroli S. (2021). Cognitive Telerehabilitation for Older Adults With Neurodegenerative Diseases in the COVID-19 Era: A Perspective Study. Front. Neurol..

[14] Mantovani E., Zucchella C., Bottiroli S., Federico A., Giugno R., Sandrini G., Chiamulera C., Tamburin S. (2020). Telemedicine and Virtual Reality for Cognitive Rehabilitation: A Roadmap for the COVID-19 Pandemic. Front. Neurol..

[15] Chae S.H., Kim Y., Lee K.S., Park H.S. (2020). Development and Clinical Evaluation of a Web-Based Upper Limb Home Rehabilitation System Using a Smartwatch and Machine Learning Model for Chronic Stroke Survivors: Prospective Comparative Study. JMIR mHealth uHealth.

[16] Fiani B., Siddiqi I., Lee S.C., Dhillon L. (2020). Telerehabilitation: Development, Application, and Need for Increased Usage in the COVID-19 Era for Patients with Spinal Pathology. Cureus.

